# Nutraceuticals in the Management of Dyslipidemia: Which, When, and for Whom? Could Nutraceuticals Help Low-Risk Individuals with Non-optimal Lipid Levels?

**DOI:** 10.1007/s11883-021-00955-y

**Published:** 2021-08-04

**Authors:** Arrigo F. G. Cicero, Federica Fogacci, Anca Pantea Stoian, Michal Vrablik, Khalid Al Rasadi, Maciej Banach, Peter P. Toth, Manfredi Rizzo

**Affiliations:** 1 Department of Medicine and Surgery Sciences, University of Bologna, Bologna, Italy; 2 Italian Society of Nutraceuticals (SINut), Bologna, Italy; 3 IRCCS Azienda Ospedaliero-Universitaria Di Bologna, Bologna, Italy; 4 Atherosclerosis Research Center, University of Bologna, Via Albertoni, 15, 40138 Bologna, Italy; 5 Faculty of Medicine, Department of Diabetes, Nutrition and Metabolic Diseases, Carol Davila University of Medicine and Pharmacy, Bucharest, Romania; 6 Third Department of Internal Medicine, General University Hospital and First Faculty of Medicine, Charles University, Prague, Czech Republic; 7 Medical Research Centre, Sultan Qaboos University, Muscat, Oman; 8 Department of Hypertension, Chair of Nephrology and Hypertension, Medical University of Lodz, Łódź, Poland; 9 CGH Medical Center, Sterling, IL USA; 10 Cicarrone Center for the Prevention of Cardiovascular Disease, Johns Hopkins University School of Medicine, Baltimore, MD USA; 11 Department of Health Promotion, Mother and Child Care, Internal Medicine and Medical Specialties (Promise), University of Palermo, Palermo, Italy

**Keywords:** Cholesterol, Dietary supplements, Nutraceuticals, Low-density lipoproteins, Safety, Efficacy

## Abstract

**Purpose of Review:**

The aim of this review is to summarize the available clinical efficacy and safety data related to the most studied and used lipid-lowering nutraceuticals.

**Recent Findings:**

A growing number of meta-analyses of randomized clinical trials supports the effectiveness and tolerability of some lipid-lowering nutraceuticals such as red yeast rice, plant sterols and stanols, soluble fibers, berberine, artichoke extracts, bergamot polyphenol fraction, garlic, green tea, and spiruline. No significant safety concern has been raised for the use of such products. Association of more lipid-lowering nutraceuticals and of some nutraceuticals with lipid-lowering drugs has been tested as well.

**Summary:**

Current evidence suggests that some clinically tested lipid-lowering nutraceuticals could be safely used to improve plasma lipid levels in subjects affected by mild-to-moderate dyslipidaemia with low cardiovascular risk.

## Introduction

Cardiovascular diseases (CVD) remain the leading cause of mortality worldwide [1], with atherosclerotic CVD (ASCVD) being the main cause of premature death in Europe and a major cause of disability and loss of productivity, with a huge global economic impact [2].

Several risk factors are etiologic for ASCVD. Among the modifiable ones, elevated plasma low-density lipoprotein cholesterol (LDL-C) level is the most important [3]. Many clinical trials have demonstrated that reducing LDL-C reduces risk for developing ASCVD and its clinical sequelae [4]. In a meta-analysis of over 170,000 secondary prevention statin trial participants, the Cholesterol Treatment Trialists’ Collaboration showed that for every 1 mmol/L reduction of LDL-C (~ 40 mg/dL), cardiovascular (CV) events are reduced by approximately 20% [5]. Hence, it has been estimated that every 1% reduction of LDL-C levels corresponds to a 1% reduction in CV events.

Currently, according to the severity of dyslipidemia and CV risk, treatment is based on lifestyle changes in dietary habits and physical activity coupled with pharmacological therapy [6]. Lifestyle treatment for hypercholesterolemia includes an energy-balanced Mediterranean diet, low in saturated fat (< 7% of total energy), moderate or higher intensity physical activity (≥ 150 min/week), and weight loss (5–10% of body weight) for those who are overweight or obese. Exposure to active or passive tobacco smoking must be also avoided [7]. LDL-C reductions with lifestyle improvements are usually in the range of 5 to 15%. If these reductions are maintained over a long period, this can result in meaningful CVD risk reduction [9••]. For patients with hypertriglyceridemia, lifestyle interventions, including weight loss if overweight or obese (initially targeting loss of 5–10% of body weight in order to relieve insulin resistance), physical activity (≥ 150 min/week of moderate or higher intensity activity), and restriction of alcohol, simple sugars, or refined carbohydrate intakes, can help reduce triglyceride (TG) levels [8].

Given the relatively small effect of lifestyle on improving cholesterol levels, the low level of adherence of patients to lifestyle changes long-term, and the need to reach low plasma LDL-C levels, there is a growing interest in natural compounds able to safely improve lipid patterns [9••]. A number of lipid-lowering nutraceuticals have been identified and clinically tested; however, their efficacy and tolerability are variable. In this context, we review the efficacy and safety of the most studied lipid-modifying nutraceuticals and their association in order to drive their prescription towards the most evidence-based one.

According to their mechanism of action, lipid-lowering nutraceuticals can be classified into (i) inhibitors of intestinal cholesterol absorption, (ii) inhibitors of hepatic cholesterol synthesis, and (iii) enhancers of LDL-C excretion. In addition, there are many functional food/nutritional supplements with multiple or unclear mechanisms of action.

The effects of the most evidence-based cholesterol-lowering agents are summarized in Table 1.

**Table 1:** Effects of the most evidence-based cholesterol-lowering agents by main mechanism of action

Main effect	Nutraceutical	Maximum reported reduction on LDL-C	Other effects
Absorption inhibitors	Plant sterols and stanols	− 12%	Mild reduction in TG, hsCRP
	Soluble fibers		
	Oat fibers	− 12%	
	Psyllium	− 7%	Mild glucose reduction
	Glucomannan	− 5%	Relevant TG reduction
	Probiotics	− 5%	Improvement in bowel function and immunity
LDL synthesis inhibition	Red yeast rice	− 20%	hsCRP reduction Mild TG reduction Mild HDL-C increase Endothelial function and arterial stiffness improvement
	Garlic	− 5%	Blood pressure decrease Antiaggregant effect
	Bergamot	− 15%	Mild TG reduction Fasting plasma glucose, insulin leptin, leptin/adiponectin, inflammatory biomarkers’ reduction
	Artichoke	− 10%	Mild TG, liver transaminase and fasting plasma glucose reduction
LDL excretion improvers	Berberine	− 15%	Mild TG reduction Mild blood pressure, fasting plasma glucose, HOMA-index, inflammatory biomarkers’ reduction
	Green tea extracts	− 5%	Mild blood pressure decrease Improvement of endothelial function and arterial stiffness
	Soy proteins	− 5%	Mild TG reduction

*HDL-C*, high-density lipoprotein cholesterol; *HOMA*, homeostasis model assessment; *hsCRP*, high-sensitivity C-reactive protein; *LDL-C*, low-density lipoprotein cholesterol; *TG*, triglycerides

## Inhibitors of Intestinal Cholesterol Absorption

### Plant Sterols and Stanols

Plant sterols, present in almost all vegetable sources (in particular in vegetable oils, nuts, seeds, legumes, and fat spreads), are structurally similar to cholesterol. Plant sources contain also plant stanols. PS (plant sterols + stanols) average daily intake in a common diet is usually less than 500 mg [10].

The main mechanism by which PS reduce LDL-C level is the decrease of intestinal absorption of exogenous cholesterol micelles in the gastrointestinal lumen interacting with the brush border membrane and substrate of the Niemann-Pick C1-Like 1 (NPC1L1) transporter [11].

A large meta-analysis of 124 studies (201 strata) concluded that the PS are safe and that their LDL cholesterol-lowering effect continues to increase up to intakes of approximately 3 g/day with an average LDL-C reduction of 12% [12]. Moreover, PS could have some impact also on TG but only in patients with high TG levels at baseline [13]. A further meta-analysis of 15 randomized clinical trials (RCTs) involving a total of 500 participants showed that stanol- or sterol-enriched diets in combination with statins, compared with statins alone, produced significant reductions in LDL-C of 0.30 mmol/L [95% confidence interval (CI) − 0.35 to − 0.25], but no change in high-density lipoprotein cholesterol (HDL-C) or TG [14].

### Soluble Fibers

Dietary fiber is a term commonly used for a variety of substances of vegetable origin resistant to enzymatic digestion in the gastrointestinal tract. Some studies demonstrated the lipid-lowering properties of soluble fibers, including pectin, guar gum, mucilages, oats, and psyllium [15•]. The lipid-lowering mechanisms of action of soluble fibers are different, including prolonged gastric emptying time, an increase of satiety, the inhibition of hepatic cholesterol synthesis, and an increase of fecal excretion of cholesterol and bile salts [16]. The reduction of cholesterolemia obtained by soluble fibers is variable and dependent on the type of fiber, doses, subjects treated, study size, and different diets: 3 g soluble fiber from oats (3 servings of oatmeal, 28 g each) can decrease LDL cholesterol by approximately 0.13 mmol/L [16]. However, the effect could be larger for oat-based fibers, psyllium, and glucomannan [16].

### β-Glucan

International guidelines for the management of dyslipidaemia suggest the consumption of 5–15 g/day (European guidelines) or 10–25 g/day (US guidelines) of soluble fibers derived from oats, rich in β-glucan to reduce blood cholesterol [17, 18]. β-Glucan is a soluble fiber derived from the walls of different plant cells, bacteria, algae, fungi, and yeasts. β-Glucan has high viscosity, which confers lipid-lowering action. A meta-analysis of 17 RCTs with 916 patients showed that β-glucan consumption in hypercholesterolemic patients significantly reduced LDL-C (− 0.21 mmol/L [8.1 mg/dL] (95% CI: 0.27; − 0.14), *p* < 0.00001). However, there were no significant differences in HDL-C and TG [19]. In a recent trial, the addition of oat fibers to a Mediterranean diet induced an LDL-C reduction of 15.1% (95% CI: − 17.8 to − 5.9) [20]. In 2010, the European Food Safety Authority (EFSA) confirmed that oat β-glucan is able to reduce plasma cholesterol levels; however, at least 3 g/day of β-glucan is necessary [21].

### Psyllium

Psyllium is a natural source of concentrated fibers derived from the husks of blonde psyllium seed. The mechanisms of action of psyllium are similar to those of other fibers already discussed, including an increased excretion of bile acids (stimulating 7-α-hydroxylase) and a reduced absorption of intestinal cholesterol [22].

A meta-analysis of 21 studies, which enrolled a total of 1030 and 687 subjects receiving psyllium or placebo, respectively, concluded that compared with placebo, consumption of psyllium lowered serum LDL-C by 0.28 mmol/L (10.8 mg/dL) (95% CI: 0.21; 0.31 mmol/L). With random-effect meta-regression, a significant dose–response relationship was found between doses (3–20.4 g/day) and LDL-C changes. Following an average intake of psyllium of 10 g/day, an average reduction of LDL-C of 7% was observed [23]. The reduction of cholesterol was more pronounced in American subjects with hypercholesterolemia, who consumed a high fat diet (LDL-C − 8/20%) [24].

### Glucomannan

Glucomannan is a soluble fiber derived from *Amorphophallus konjac* (konjac root) [25]. Unlike other fibers, glucomannan does not act by binding bile acids, but it seems to reduce the absorption of cholesterol in the jejunum and the absorption of bile acids in the ileum. Moreover, it increases the activity of 7-α-hydroxylase converting cholesterol into bile acids [25].

A meta-analysis of 14 RCTs with 531 patients concluded that the use of glucomannan (at doses ranging between 1.2 and 15.1 g/day) significantly reduces LDL-C and TG respectively by − 0.41 mmol/L (15.9 mg/dL) and − 0.13 mmol/L (11.5 mg/dL) (*p* < 0.05 for both), compared to placebo. The reduction of serum TG is a peculiarity of glucomannan probably due to its high viscosity and its ability to interfere with hepatic cholesterol and lipoprotein metabolism [26].

### Probiotics

Probiotics are defined as vital microorganisms, which confer health benefits to the host when taken in adequate amounts [27]. In recent years, some clinical studies have supported the hypothesis of a possible clinical use of certain strains of microorganisms as cholesterol-lowering agents. Nevertheless, it is still difficult to draw firm conclusions due to the great heterogenity of the studies in terms of duration of treatment, type of probiotic strains used, dosage, clinical characteristics of the participants, and dosage form/vehicle.

Among the proposed lipid-lowering mechanisms, it is possible that probiotics interact with the intestinal cholesterol, binding or incorporating it into the bacteria cell membranes [28]. A meta-analysis has included 30 RCTs to investigate the effect of probiotics on total cholesterol (TC), HDL-C, and TG and 27 RCTs on LDL-C. The most studied probiotic strains were *Lactobacillus acidophilus*, *Lactobacillus acidophilus* + *Bifidobacterium lactis*, and *Lactobacillus plantarum*. The pooled mean net change in LDL-C was of − 0.19 mmol/L (7.35 mg/dL) (*p* < 0.01) compared to controls. TG and HDL-C did not change significantly compared to the control groups [29]. The best results were obtained with *Lactobacillus* strains [30]: one possible explanation might be the adaptation of *Lactobacillus* (in particular *Lactobacillus acidophilus* and *Lactobacillus plantarum*) that can survive in an acid- and bile-rich environment and easily colonize the gastrointestinal tract [31]. Probiotics are considered to be generally safe.

## Inhibitors of Liver Cholesterol Synthesis

### Red Yeast Rice Extract

Red yeast rice (RYR) is a nutraceutical obtained by the fermentation of a particular yeast (in general *Monascus purpureus*) in white rice; the red coloration is produced by pigments resulting from secondary fermentative metabolism [32]. The yeast during the fermentation process enriches the rice with a complex of substances with important lipid-lowering activities including polyketides as monacolins [33•]. Based on the conditions of fermentation and the yeast strain used, today several types of monacolins have been identified including the subtype monacolin K (MonK) structurally identical to lovastatin, whose main cholesterol-lowering mechanism of action of red yeast rice is reversible inhibition of 3-hydroxy-3-methyl-glutaryl-CoA (HMG-CoA) reductase.

Despite the same structure, MonK and lovastatin pharmacokinetic profiles and bioavailability are different. In fact, if lovastatin is administered in conventional pharmaceutical form as a single active ingredient (31% of bioavailability in humans), MonK is only one of the components of the RYR that can interact changing the typical pharmacokinetic profile of lovastatin [34, 35].

A meta-analysis of 20 RCTs evaluated the efficacy of RYR supplementation after 2–24 months that reduced LDL-C on average of 1.02 mmol/L (− 1.20; − 0.83) (39.4 mg/dL) compared to placebo which was not different from moderate-intensity statins (pravastatin 40 mg, simvastatin 10 mg, lovastatin 20 mg) (0.003 mmol/L; − 0.36; 0.41) (0.12 mg/dL). A small increase of HDL-C (0.007 mmol/L; 0.03; 0.11) (0.3 mg/dL) and decrease of TG (− 0.26 mmol/L; − 0.35; − 0.17) (23 mg/dL) compared to placebo were observed as well. The doses of RYR used were different and varied from 1200 to 4800 mg/day containing from 4.8 to 24 mg of MonK [36].

Some trials show that RYR also improves inflammatory biomarkers, endothelial function (flow-mediated dilation), and carotid-femoral pulse-wave velocity in humans [37••]. RYR use is a rare example of a nutraceutical studied to evaluate its effects on CV outcomes. RYR supplementation has shown efficacy in reducing CVD risk in adult and elderly patients in secondary prevention [38]. In a large trial involving 66 hospitals in China, 1445 patients (aged between 65 and 75 years) with a history of myocardial infarction were randomized into two groups (placebo vs*.* RYR) and followed for a mean of 4 years. RYR supplementation showed a reduction in the risk of CHD (31.0%, *p* = 0.04), all-cause mortality (31.9%, *p* = 0.01), stroke (44.1%, *p* = 0.04), the need for coronary artery bypass graft or a percutaneous coronary intervention (48.6%, *p* = 0.07), and malignancies (51.4%, *p* = 0.03). It has also been estimated that following a RYR treatment during 4 years, the numbers needed to treat to prevent one coronary event, one coronary death, and one mortality due to all causes in elderly patients were 18, 33, and 23, respectively. Side effects were not significantly different in the two groups [46].

Inhibitors or inducers of cytochrome P450 (CYP450) may cause alterations of plasma concentrations of MonK. In fact, the concomitant use of some nutraceuticals (such as grapefruit juice) [39], food, or drugs being CYP450 inhibitors may increase the risk of myotoxic side effects and in some rare cases cause rhabdomyolysis [40]. Attention must be made to citrinin, a mycotoxin metabolite derived from the fermentation of *Monascus* [41]. The chronic ingestion of citrinin is nephrotoxic in various animal species, gradually leading to hyperplasia of the renal tubular epithelium, renal adenomas, and in some cases malignant renal tumors. Moreover, citrinin induces reproductive toxicity, malformations, and proven embryo toxicity in vitro and in vivo, so that the EFSA has defined 0.2 µg/kg b.w. per day as the highest quantity of citrinin which could be taken by humans with no nephrotoxic effects [42]. In the market, RYR supplements were detected with levels of citrinin exceeding 114 µg/capsule, and for 4 capsules/day (recommended dosage), the mean was 456 µg/day of citrinin, which is well above the level of 20 µg/kg b.w. per day suggested by the EFSA [43].

Moreover, a recent meta-analysis of 53 RCTs comprising 112 treatment arms, which included 8535 subjects, with 4437 in the RYR arm and 4303 in the control one, showed that MonK administration was not associated with increased risk of muscle-related side effects (odds ratio (OR) = 0.94, 95% confidence interval (CI) 0.53,1.65). Moreover, reduced risk of non-muscle-related side effects (OR = 0.59, 95% CI 0.50, 0.69) and serious adverse events (OR = 0.54, 95% CI 0.46, 0.64) versus control was shown [44••].

### Garlic

Allicin produced from the non-proteinogenic amino acid alliin in a reaction catalyzed by alliinase is responsible for the lipid-lowering effect of garlic (*Allium sativum*) [45]. In fact, allicin is an inhibitor of HMG-CoA reductase, squalene-monooxygenase, and acetyl-Coenzyme A (acetyl-CoA) synthetase. Another possible mechanism by which garlic may act is the promotion of bile acid excretion [46].

In a meta-analysis of 39 RCTs enrolling 2298 mild-to-moderate hypercholesterolemic subjects, the consumption of garlic extracts for at least 2 months showed a reduction of LDL-C (− 0.23 mmol/L [9 mg/dL], more evident in individuals with TC < 5.17 mmol/L [200 mg/dL] at baseline) [47]. The same author highlighted the beneficial effect on blood pressure of garlic [48]. Side effects are usually minimal (mostly gastrointestinal) and the extracts are well tolerated [46].

### Bergamot (Citrus bergamia)

Bergamot is the common name of the fruit *Citrus bergamia Risso* and differs from other *Citrus* fruits for its composition, particularly rich in flavonoids (as neoeriocitrin, neohesperidin, naringin, rutin, neodesmin, rhoifolin, poncirin) [49]. In particular, the 3-hydroxy-3-methyl-glutaryl flavanones enriched fraction (brutieridin, melitidin, and neoeriocitrin) has been extracted from the bergamot peel, which act as statins by inhibiting HMG-CoA reductase and Acyl-CoA cholesterin acyltransferase (ACAT), reducing the formation of cholesterol esters and apoB lipoproteins. Bergamot contains also naringin: like neoeritrocitrin, meltidin and rutinit, it inhibits the oxidation of LDL-C and activates adenosine-monophosphate-kinase (AMPK). It is also possible that bergamot increases the fecal excretion of cholesterol, and reduces the intestinal absorption and increases the turnover and excretion of bile acids [50, 51].

Clinical studies on the lipid-lowering properties of bergamot show that bergamot-derived polyphenols (500 to 1500 mg/day) are able to reduce LDL-C, triglycerides, non-HDL-C, malonyl dialdehyde [52], fasting plasma insulin, leptin, leptin/adiponectin ratio, hs-CRP, and tumor necrosis factor alpha (TNF-α) [53], in a dose-dependent manner, largely variable depending on the degree of extract purification.

### Artichoke

According to some clinical investigations, the artichoke leaf extract (*Cynara scolymus*, *Cynara cardunculus*) has potential hypolipidemic and hepatoprotective effects, mainly attributed to mono- and dicaffeoylquinic acid (cynarin and chlorogenic acid), caffeic acid, volatile sesquiterpene, and flavonoids. The lipid-lowering mechanisms of artichoke seem to be essentially two—the interaction of luteolin with the HMG-CoA reductase and the pathways of regulation in the liver of sterol regulatory element-binding proteins (SREBPs) and ACAT [54].

A meta-analysis of nine RCTs with 702 subjects suggested a significant decrease in plasma concentrations of TC [weighted mean difference (WMD): − 17.6 mg/dL (0.46 mmol/L), 95% CI: − 22.0, − 13.3, *p* < 0.001], LDL-C [− 14.9 mg/dL (0.39 mmol/L), 95% CI: − 20.4, − 9.5, *p* = 0.011], and triglycerides [WMD: − 9.2 mg/dL (0.1 mmol/L), 95% CI: − 16.2, − 2.1, *p* = 0.011] [55]. In addition, artichoke extract showed pleiotropic effects mildly improving liver transaminases, fasting blood sugar, and systolic blood pressure [56]. In the available studies, no side effect has been detected beyond minor and transient gastrointestinal ones.

## Inducers of LDL Cholesterol Excretion

### Berberine

Berberine (BBR) is a quaternary benzylisoquinoline alkaloid present in the root, rhizome, stem, fruit, and bark of different species of plants such as *Coptis* (*Coptis chinensis*, *Coptis japonica*), *Hydrastis* (*Hydrastis canadensis*), and *Berberis* (*Berberis aristata*, *Berberis vulgaris*, *Berberis croatica*) [57]. BBR is an inhibitor of proprotein convertase subtilisin/kexin type 9 (PCSK9) through the ubiquitination and degradation of hepatocyte nuclear factor 1 alpha (HNF-1α), causing increased levels and a reduction in the lysosomal degradation of hepatic LDL receptors (LDLR). BBR also acts directly on the expression of LDLR via two identified mechanisms, causing an upregulation of the receptors through a post-transcriptional mechanism that stabilizes LDLR messenger ribonucleic acid (mRNA) [58]. Recent studies have shown that BBR reduces also the intestinal absorption of cholesterol, increasing fecal excretion and promoting hepatic cholesterol turnover and the formation of bile acids [59]. Moreover, BBR is an activator of AMPK, which activates fatty acid oxidation and inhibits the expression of lipogenic genes [60]. Finally, it is an effective inhibitor of nicotinamide adenine dinucleotide phosphate (NADPH) oxidase-mediated oxidative stress [61]. The bioavailability of BBR is lower than 1%: this is due to (1) poor intestinal absorption (56%), (2) low permeability of the molecule, and (3) the intestinal and liver first-pass metabolism (43.5% and 0.14%, respectively) [59].

The lipid-lowering efficacy of BBR has been confirmed by a meta-analysis that included 27 clinical studies with 2569 participants: LDL-C: − 0.65 mmol/L (95% CI − 0.75; − 0.56, *p* = 0.00001) (25.14 mg/dL); TG: − 0.39 mmol/L (95% CI − 0.59; − 0.19, *p* = 0.0001) (34.5 mg/dL); HDL-C: 0.07 mmol/L (95% CI 0.04; 0.10, *p* = 0.00001) (2.71 mg/dL). These effects seem to be additive to those of statins and associated with a positive impact also on glucose metabolism and blood pressure [62]. A recent study enrolled 130 patients undergoing percutaneous coronary intervention (PCI), randomized into two groups, and treated with BBR 600 mg/day or placebo in addition to standard therapies. The BBR group showed a marked reduction in TG (26% BBR vs. 13% control: the difference did not reach statistical significance due to large inter-individual variations) and LDL-C (24% vs. 17% BBR control: *p* < 0.001) compared to the control group. In addition, both groups showed a reduction in the levels of interleukin 6 (IL-6) and monocyte chemoattractant protein-1 (MCP-1) (*p* < 0.05 for each), as well as hsCRP, intercellular adhesion molecule-1 (ICAM-1), vascular cell adhesion molecule-1 (VCAM-1), and matrix metallopeptidase 9 (MMP-9) (*p* < 0.001 for all) compared to baseline [63].

Side effects from BBR are mild to moderate, mostly gastrointestinal (diarrhea, constipation, abdominal distension) and comparable in frequency to the control groups. No significant differences were detected in the levels of liver alanine transaminases and creatinine in comparison to the control group [64].

### Green Tea Extracts

Green tea is particularly rich in polyphenol antioxidants [65]. The major fraction of polyphenols in green tea is catechins including epigallocatechin-3-gallate (EGCG). It is possible that beyond the antioxidant effects derived from polyphenols and the reduction of lipid peroxidation, green tea interferes with micellar solubilization and absorption of cholesterol. Green tea is an activator of AMPK (stimulating lipogenesis) and also an inhibitor of HMG-CoA reductase. Tea catechins have been reported to have an inhibitory action on the ileal apical sodium-dependent bile acid transporter (reducing reabsorption of bile acids) and to enhance hepatic LDLR expression and the biliary excretion of cholesterol [66, 67].

A meta-analysis of 20 RCTs and 1536 participants showed a reduction of LDL-C [mean difference (MD): − 0.19 mmol/L; 95% CI: − 0.3; − 0.09, *p* = 0.0004]. Moreover, green tea extract exerts a mild but significant antihypertensive effect, an improvement in flow-mediated dilation (FMD) [68] and pulse wave velocity (PWV) [69]. The tested daily doses ranged from 250 to 1200 mg of green tea extract or from 170 to 850 mg of EGCG [70]. Green tea is well tolerated. High doses of green tea can cause a deficiency of iron and folate due to its capacity to bind and reduce their intestinal absorption. Therefore, particular attention should be given to green tea consumption during pregnancy [70].

### Soy Proteins

Preclinical and clinical evidence supports the positive effects of soy proteins on the lipid profile. Bioactive peptides present in soy (i.e., isoflavones) could contribute to this effect [71]. The cholesterol-lowering mechanisms proposed for soy seem to be numerous: there is downregulation of the expression of the hepatic transcription factor SREBP-1 via the phosphatidylinositol 3-kinase/protein kinase B/glycogen synthase kinase-3β (PI3K/Akt/GSK3β) pathways (with decreased hepatic lipoprotein secretion and cholesterol content), activation of SREBP-2 (with increased LDLR expression and clearance of cholesterol from the blood), reduction of cholesterol biosynthesis, and an increase in the fecal excretion of bile salts [72, 73].

A meta-analysis of 35 RCTs and 2670 subjects concluded that soy proteins have a cholesterol-lowering effect with a mean reduction in LDL-C of 3% (− 0.12 mmol/L/4.6 mg/dL) and TG of 4% (− 0.06 mmol/L/5.3 mg/dL) and are able to increase HDL-C by 3% (+ 0.04 mmol/L/1.6 mg/dL), the effect being proportional to the baseline LDL-C level [74]. The mean tested dose was 30 g/day. If isoflavones seem not to add significantly to the lipid-lowering effect of soy proteins, they seem to have direct positive effects on endothelial function [75] and arterial stiffness [76].

The chronic use of high quantities of soy products containing isoflavones could interfere with thyroid function and fertility. Furthermore, soybean and its derivatives contain high amounts of phytic acid that reduces the absorption of minerals such as calcium, magnesium, copper, iron, and zinc. The large amount of vegetable proteins, which have to be taken in order to obtain a significant LDL-C reduction, could decrease patient compliance in the long-term and should be accompanied by an attentive balance to the other dietary sources of proteins.

## Other Lipid-Lowering Nutraceuticals with Mixed Mechanisms of Action

### Polyunsaturated Omega-3 Fatty Acids

Omega-3 (ώ-3) fatty acids are polyunsaturated fatty acids (PUFAs), which contain a double bond in position 3 at the end of the carbon chain. Natural sources of omega-3 are present both in animal (fish, krill, egg, squid) and plant (algae, flaxseed, walnut, edible seeds, clary sage, seed) sources [77]. In recent years, the EFSA, the American Heart Association (AHA), and the Food Standards Australia and New Zealand (FSANZ) organizations have recognized the omega-3 as preventive nutraceuticals for CVD. The EFSA established a claim in 2010 indicating that the intake of at least 2 g/day of docosahexaenoic acid (DHA) and eicosapentaenoic acid (EPA) has the ability to maintain normal blood TG levels [78, 79]. The AHA has indicated doses from 2 to 4 g/day of EPA/DHA to reduce TG levels by 25–30% [80]. All these guidelines agree about high safety of PUFAs, apart from relatively frequent fishy aftertaste and rare abdominal discomfort.

The mechanisms through which omega-3 reduce TG are the following: (1) reduction synthesis of hepatic very-low-density lipoprotein (VLDL), (2) reduction of available substrate for the synthesis of new TG (omega-3 s are false substrates), (3) reduction of in the activity of TG-synthesizing enzymes (diacylglycerol acyltransferase or phosphatidic acid phosphohydrolase), (4) increase of fatty acid β-oxidation, and reduction of the endogenous synthesis of fatty acids and the increased of synthesis of phospholipids [81].

A meta-analysis of Eslick et al. included 47 RCTs with 16,511 participants with hypercholesterolemia to assess the effects of the average daily dose of 3.25 g of EPA/DHA for 24 weeks. The results showed a significant reduction in TG of 14% [− 0.34 mmol/L (30.12 mg/dL), 95% CI: − 0.41; − 0.27 mmol/L (− 36; − 24 mg/dL)]. In addition, there was a small insignificant reduction of LDL-C of 0.06 mmol/L (2.3 mg/dL), but no differences in HDL-C [82]. PUFAs might also improve FMD, PWV, and, with larger dosages, positive effects on inflammatory diseases and mood [83].

A rich source of omega-3 PUFAs is the krill (*Euphausia superba*), a small crustacean that lives in the Antarctic Ocean, containing many types of long-chain PUFAs. The omega-3 present in the krill oil (EPA + DHA) appear to be better absorbed in the gastrointestinal tract than those found in fish oil: this is possible because of phosphatidylcholine (the main phospholipid present in the krill (40%) that binds EPA and DHA) that confers a greater stability to fatty acids. In addition, krill oil is rich in antioxidants including vitamin E and astaxanthin [84]. Therefore, at the same dose, krill oil appears to be more effective than fish oil in the adjustment of the lipid profile [85].

In a meta-analysis of 7 RCTs with 662 participants, Ursoniu et al. showed a significant reduction in plasma concentrations of LDL-C [− 0.4 mmol/L (− 15.52 mg/dL); 95% CI: − 0.73; − 0.07 mmol/L (− 28.43; − 2.61 mg/dL); *p* = 0.018], TG [− 0.16 mmol/L (− 14.03 mg/dL); 95% CI: − 0.24; − 0.08 mmol/L (− 21.38; − 6.67 mg/dL)], and significant elevation in plasma concentrations of HDL-C [0.17 mmol/L (6.65 mg/dL); 95% CI: 0.06; 0.28 mmol/L (2.30; 10.99 mg/dL)] following supplementation with krill oil [86].

Controversial results were obtained with the alpha-linolenic acid (ALA), an omega-3 found in many vegetable oils (such as olive and flaxseed oil). Nevertheless, a rich source of ALA, the flaxseed (*Linum usitatissumum*, ALA = 50–62% of flaxseed oil or 22% of whole flaxseed), an oilseed crop grown on all continents, showed a lipid-lowering activity regarding LDL-C (− 0.08 mmol/L): this effect may be explained by the other components of flaxseed such as lignans (0.2–13.3 mg/g flaxseed) and soluble fibers (25% of total weight) that could enhance the reduction of serum cholesterol. The cholesterol-lowering effects were more significant in females (in particular in postmenopausal women) and in individuals with high cholesterol levels at baseline [87]. The available data suggest that the consumption of flaxseed is safe and well tolerated.

### Spirulina

Spirulina (*Arthrospira platensis*) is a filamentous microalga with known lipid-lowering effects, but with an unclear mechanism of action [88]. Spirulina contains high amounts of antioxidants and PUFAs. C-phycocyanin, a particular essential pigment of spirulina, contains a phycocyanobilin, which can activate atheroprotective heme oxygenase-1 (HMOX-1), a key enzyme in the heme catabolic pathway in endothelial cells. Moreover, phycocyanin has proven antioxidant, anti-inflammatory, and radical scavenging properties.

According to experimental studies in alloxan-injured mice, phycocyanin decreases TC and TG levels in serum, increases the hepatic glycogen level, and maintains glucokinase expression in the liver. A recent meta-analysis has included seven clinical trials to assess the effect of spirulina supplementation on plasma lipid concentrations showed the lipid-lowering efficacy of spirulina, with a reduction of LDL-C by − 1.1 mmol/L (95% CI: − 1.6; − 0.6, *p* < 0.001), TC by 1.2 mmol/L (95% CI: − 1.7; − 0.7, *p* < 0.001), and TG by − 0.5 mmol/L (95% CI: − 0.6; − 0.4, *p* < 0.001) and an increase of HDL-C of + 0.16 mmol/L (95% CI: 0.06; 0.25, *p* = 0.001) [89].

## Nutraceutical Combinations

**Fig. 1:**
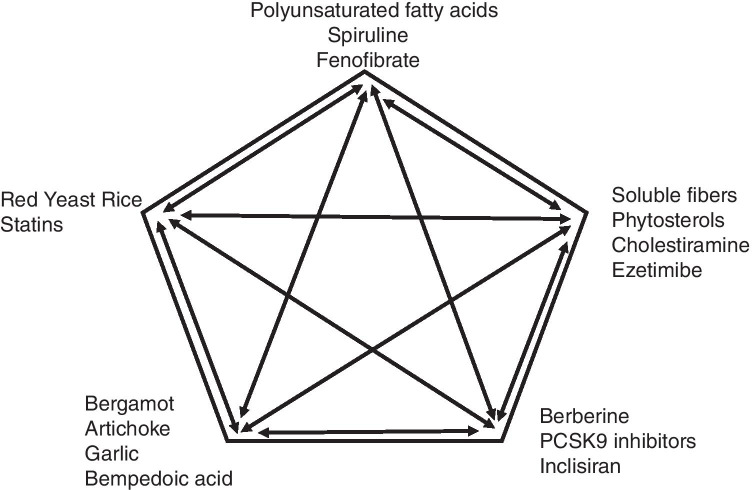
Possible lipid-lowering nutraceuticals and drugs association to improve plasma lipid control in humans

Rational combinations of nutraceuticals with different lipid-lowering activities (Fig. 1), particularly when associated with an appropriate lifestyle, can provide an alternative to drug treatment in patients in primary CVD prevention with mildly elevated LDL-C (especially for those not being at their target LDL-C)) and in some statin-intolerant patients [90]. There are many nutraceuticals with significant lipid-lowering properties: most of them are used in combination with a low dosage of other nutraceuticals, statins, and other lipid-lowering drugs, because that permits one to reduce the risk of side effects and to improve the efficacy (reduce the residual CV risk) [91]. Moreover, natural products with different mechanisms of action may have synergetic effects, acting on the absorption of lipids from the bowel and/or increasing their excretion (soluble fibers, glucomannan, plant sterols, probiotics), enhancing the hepatic uptake of cholesterol (berberine, soybean proteins), inducing LDL-C excretion (berberine, soy proteins, chlorogenic acid), inhibiting HMG-CoA reductase enzyme and limiting the hepatic synthesis of cholesterol (monacolins, policosanols, allicin, soybean proteins, bergamot), reducing the oxidation of the LDL, and increasing thermogenesis and lipid metabolism (chlorogenic acid) [92].

Below, we focus on some combined lipid-lowering nutraceuticals whose efficacy and safety have been confirmed by more than one randomized clinical trial.

The lipid-lowering properties of RYR (3 mg MonK), policosanols (10 mg), and berberine (500 mg) are the most studied in RCT and the only one for which meta-analyses of RCTs are available. A recent meta-analysis of 12 randomized double-blind placebo-controlled clinical trials including 1050 subjects suggested that dietary supplementation with this nutraceutical combination exerts a significant effect on body mass index (mean difference (MD) =  − 0.25 kg/m^2^, *p* = 0.008) and serum levels of LDL-C (MD =  − 26.67 mg/dL, *p* < 0.001), TG (MD =  − 11.47 mg/dL, *p* < 0.001), HDL-C (MD = 1.84 mg/dL, *p* < 0.001), high-sensitivity C-reactive protein (hs-CRP; MD =  − 0.61 mg/L, *p* = 0.022), and fasting glucose (MD =  − 3.52 mg/dL, *p* < 0.001) [93]. This combination has also been tested in adult and elderly patients previously intolerant to statins [94, 95], being well tolerated in the 80% of cases. Based on the available data, no serious safety concerns have been observed.

The association of plant sterols/stanols with certain lipid-lowering ingredients also demonstrated to strengthen cholesterol-lowering efficacy and add TG-lowering effects [96]. Moreover, some available trials have been carried out on an association of RYR (166.67 mg, 0.4% monacolin K), sugar cane-derived policosanols (3.70 mg), and artichoke leaf extracts (200 mg, 5–6% chlorogenic acid) to be taken 3 tablets per day. In a double-blind, randomized, parallel controlled study on 39 subjects with moderate hypercholesterolemia, after 16 weeks of treatment, LDL-C was reduced by 21.4% (95% CI: − 13.3: − 24.9%, *p* < 0.001), while TG decreased by 12.2% (95% CI: − 24.4; − 0.1%, *p* < 0.05) [97]. Doubling the daily dose seemed not to add additional benefits, while there were no safety concerns also with the higher dosage [98].

Recently, the association of RYR (200 mg, containing monacolin K 10 mg), artichoke extract (500 mg), and banaba extract (50 mg) has been evaluated in a double-bind, placebo-controlled, cross-over designed trial in 30 adults with suboptimal LDL-C levels, in primary prevention of CVD. Patients followed a period of 6 weeks of treatment with a nutraceutical or placebo, then 2 weeks of washout, and finally 6 weeks in crossover. After the active treatment, there was a significant improvement in LDL-C (− 18.2%), non-HDL-C (− 15%), glutamic oxaloacetic transaminase (− 10%), glutamate-pyruvate transaminase (− 30.9%), and hs-CRP (− 18.2%) versus placebo [99]. Based on the available data, no serious safety concerns have been observed.

## Discussion

A large number of nutraceuticals have been tested in available trials demonstrating their lipid-lowering effects. It is, however, important to once again emphasize that nutraceuticals cannot replace lipid-lowering therapy but might help to optimize it (reducing CV residual risk). Taking into account the influence of some of the presented nutraceuticals on different lipid parameters, it seems that this therapy might be especially important to be considered for patients with mixed dyslipidemia, especially atherogenic dyslipidemia in patients with diabetes and metabolic syndrome, in patients with low to moderate hypercholesterolemia not at LDL-C goal, and in all patients with statin-associated side effects, which are not able to be treated with statins/suitable doses of statins and are at the higher risk of CV events [100, 101••].

However, the main concern is still which lipid-lowering effects of nutraceuticals are clinically relevant, which are maintained in the long-term, and which might be associated with an improvement in CVD risk. Combinations of lipid-lowering nutraceuticals could improve their safety (reducing the dosages of the single components) but their efficacy has been rarely tested in more than one study/RCT, while some of the tested nutraceutical combinations contained underdosed components. On the other hand, both single components and some combinations (in particular red yeast rice, berberine, policosanol combination) have been demonstrated to maintain their efficacy in the long term (years), to have a positive impact on CVD risk factors other than LDL-C, and to improve some markers of vascular aging (endothelial function, pulse wave velocity). Finally, some nutraceuticals have shown to significantly improve the efficacy of standard pharmacological treatments. In this context, an evidence-based approach to the use of lipid-lowering nutraceuticals could improve the quality of the treatment, including therapy adherence, and the achievement of LDL-C goal in clinical practice. However, it has to be clearly stressed that there is still no outcome studies proving that nutraceuticals can prevent CVD morbidity or mortality in a primary prevention setting.

Even knowing that statins are the drugs of choice in patients with high LDL-C levels and moderate to high CV risk, the use of high-intensity statins increases side effects and these are associated with reduced therapy adherence and compliance [102, 103]. On the other side, even with good statin therapy tolerability, the LDL-C targeted levels migh be not achieved for 30–70% patients (depending on the risk), even in combination with ezetimibe for high-risk and very high-risk patients [104]. Many nutraceutical options are available either alone or in combination with statins to help to reach recommended goals in a safe and tolerable way for most patients [105]. Clinical trials have reported that many nutraceuticals have an additive effect on lipid-lowering drugs, allowing reduction of the statins’ doses without diminishing the results in terms of TC and LDL-C reduction and significantly limiting adverse effects [106••]. However, most of the presented combinations have been tested only in a single study and the obtained results have not been confirmed yet, and it does not allow making any recommendation. Some evidence of additive efficacy is available for the association of statins with PUFAs, soluble fibers, plant sterols, bergamot, tocotrienols, garlic, and vitamin D [107]. Ezetimibe efficacy has been improved by the association with RYR, policosanol, and berberine [108, 109].

If sufficient progress is not made towards achieving atherogenic cholesterol goals, consideration may be given to the use of lipid-lowering nutraceuticals, alone or in the combination with pharmacological therapy, which are indicated for patients with borderline lipid values ( above target) or intolerant to drugs.

## Conclusion

The use of some lipid-lowering nutraceuticals (and their association) is supported by preclinical and clinical evidence of efficacy and safety. However, the use of nutraceuticals should never substitute the one of conventional drugs, when their prescription is indicated by the international guidelines.

## Abbreviations

ACAT: Acyl-CoA cholesterin acyltransferase
Acetyl-CoA: Acetyl-Coenzyme A
AHA: American Heart Association
ALA: Alpha-linolenic acid
AMPK: Adenosine-monophosphate-kinase
ASCVD: Atherosclerotic cardiovascular diseases
BBR: Berberine
CI: Confidence interval
CV: Cardiovascular
CVD: Cardiovascular diseases
CYP450: Cytochrome P450
DHA: Docosahexaenoic acid
EFSA: European Food Safety Authority
EGCG: Epigallocatechin-3-gallate
EPA: Eicosapentaenoic acid
FMD: Flow-mediated dilation
FSANZ: Food Standards Australia and New Zealand
HDL-C: High-density lipoprotein cholesterol
HMG-CoA: 3-Hydroxy-3-methyl-glutaryl-CoA
HMOX-1: Heme oxygenase-1
HNF-1α: Hepatocyte nuclear factor 1 alpha
HOMA: Homeostasis model assessment
hsCRP: High-sensitivity C-reactive protein
ICAM-1: Intercellular adhesion molecule-1
IL-6: Interleukin 6
LDL-C: Low-density lipoprotein cholesterol
LDLR: LDL receptor
MCP-1: Monocyte chemoattractant protein-1
MD: Mean difference
MMP-9: Matrix metallopeptidase 9
MonK: Monacolin K
mRNA: Messenger ribonucleic acid
NADPH: Nicotinamide adenine dinucleotide phosphate
NPC1L1: Niemann-Pick C1-Like 1
OR: Odds ratio
PS: Plant sterols
PCI: Percutaneous coronary intervention
PCSK9: Proprotein convertase subtilisin/kexin type 9
PI3K/Akt/GSK3β: Phosphatidylinositol 3-kinase/Protein kinase B/Glycogen synthase kinase-3β
PUFAs: Polyunsaturated fatty acids
PWV: Pulse wave velocity
RCTs: Randomized clinical trials
RYR: Red yeast rice
SREBPs: Sterol regulatory element-binding proteins
TC: Total cholesterol
TG: Triglycerides
TNF-α: Tumor necrosis factor alpha
VCAM-1: Vascular cell adhesion molecule-1
VLDL: Very-low-density lipoprotein
WMD: Weighted mean difference
